# The Dark Side of Activated Phosphoinositide 3-Kinase-δ Syndrome 2: A Story Rewritten through FDG-PET

**DOI:** 10.3390/jcm13082203

**Published:** 2024-04-11

**Authors:** Arianna Catelli, Cristina Nanni, Rita Mulè, Pier Luigi Zinzani, Elena Sabattini, Marcello Lanari, Francesca Conti

**Affiliations:** 1 Specialty School of Paediatrics-Alma Mater Studiorum, University of Bologna, 40138 Bologna, Italy; 2 Nuclear Medicine, IRCCS Azienda Ospedaliero-Universitaria di Bologna, 40138 Bologna, Italy; 3 Internal Medicine and Rheumatology Unit, AUSL Bologna-IRCCS Azienda Ospedaliero-Universitaria di Bologna, 40138 Bologna, Italy; 4 IRCCS Azienda Ospedaliero-Universitaria di Bologna, Istituto di Ematologia “Seràgnoli”, 40138 Bologna, Italy; 5 Department of Medical and Surgical Sciences (DIMEC), University of Bologna, 40138 Bologna, Italy; 6 Hematopathology Unit, IRCCS Azienda Ospedaliero-Universitaria di Bologna, 40138 Bologna, Italy; 7 Pediatric Emergency Unit, IRCCS Azienda Ospedaliero-Universitaria di Bologna, 40138 Bologna, Italy; 8 Pediatric Unit, IRCCS Azienda Ospedaliero-Universitaria di Bologna, 40138 Bologna, Italy

**Keywords:** FDG-PET, immunodeficiency, APDS2, lymphoproliferation, lymphoma

## Abstract

**Background:** Activated phosphoinositide 3-kinase-δ syndrome 2 (APDS2) is characterized by lymphoproliferation and increased risk of malignancy. FDG-PET/CT may represent a helpful diagnostic tool for differentiating these clinical features and correctly diagnosing inborn errors of immunity (IEI). **Case report:** We present the case of a female patient diagnosed with Hodgkin’s lymphoma at 19 years of age, although atypical imaging aspects emerged: baseline FDG-PET/CT revealed several hot lymph nodes with a symmetrical distribution, and increased tracer uptake in spleen, axial, and appendicular bone marrow. Imaging repeated after chemotherapy and autologous stem cell transplantation showed persistent increased FDG uptake at multiple supradiaphragmatic nodes and in bone marrow. After the diagnosis of APDS2 and rapamycin treatment, FDG-PET/CT confirmed complete metabolic normalization of all sites. **Conclusions:** In the IEI scenario, FDG-PET/CT plays an effective role in differentiating malignant proliferation and immune dysregulation phenotypes. Atypical patterns at FDG-PET/CT should be interpreted as a red flag for the need of an early immunological evaluation.

## 1. Introduction

Lymphoproliferation and increased risk of malignancy are key features of activated phosphoinositide 3-kinase-δ syndrome 2 (APDS2), an inborn error of immunity (IEI) due to *PIK3R1* mutation. This generates subsequent hyperactivation of phosphatidylinositol 3-kinase delta (PI3Kδ) complex and Akt-mTOR, one of the intracellular pathways involved in differentiation, growth, and proliferation, predominantly in T- and B-cells and in neutrophils [1,2,3]. Lymphoid proliferation is a common clinical feature of APDS2, involving up to 89% of patients. Clinical manifestations are hepatosplenomegaly, lymphadenopathies, and gastrointestinal and respiratory lymphoid infiltrates [4,5]. However, increased incidence of hematologic malignancy is among the major risks of APDS1 and APDS2. B-cell lymphomas are most frequently reported, with a prevalence of Hodgkin lymphoma (HL) and diffuse large B-cell lymphoma, although marginal zone B-cell lymphoma and mucosal-associated lymphoid tissue-derived lymphoma are also observed [6]. 

Alongside immunoglobulin replacement and antibiotics, the use of rapamycin (sirolimus) was experimented with in this patient setting with promising results. It inhibits the mammalian target of rapamycin (mTOR) and influences cell proliferation. Non-neoplastic lymphoproliferation shows the best response to rapamycin treatment, as described by the European Society for Immunodeficiencies APDS registry [4]. Together with selective PI3Kδ inhibitors, these treatments are used as a bridge therapy [7] to allogenic hematopoietic stem cell transplantation (HSCT), which remains the curative option capable of phenotype reversing [8].

Differentiation between benign and malignant lymphoproliferation is essential. Indeed, there is the necessity of a less invasive assessment to discriminate between active malignancy and benign adenopathy. Imaging methods showed encouraging results in the setting of IEI, although several limitations still exist. Positron emission tomography/computed tomography (PET/CT), which uses ¹⁸F-fluorodeoxyglucose (FDG) uptake as a sensor of glucose absorption, represents the gold standard technique for both initial staging, risk stratification, and therapy assessment of lymphomas, as these cells are generally characterized by high metabolism [9]. Here, we report the successful use of FDG-PET/CT for accurate identification of APDS2-associated benign lymphoproliferation and its response to rapamycin treatment in a young patient. 

## 2. Case Description

**Figure 1:**
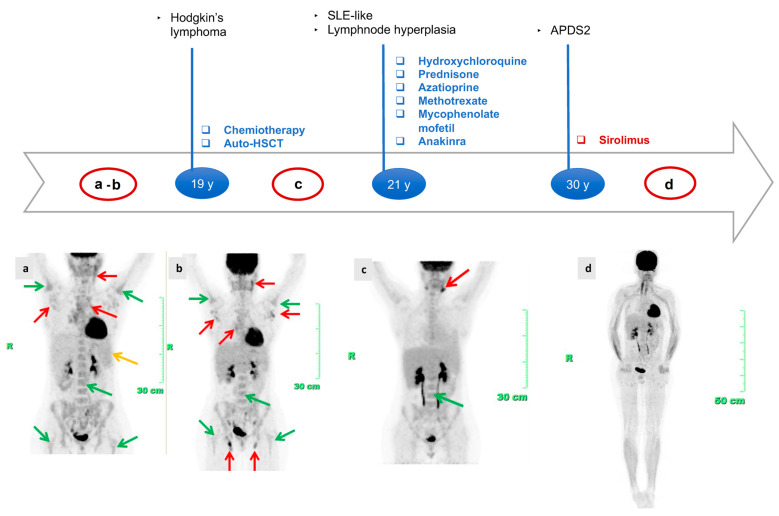
Principal clinical events, therapeutic strategies, and FDG-PET/CT timeline: (i) Hodgkin’s lymphoma treated with six cycles of doxorubicin, bleomycin, vinblastine, dacarbazine (ABVD), two cycles of ifosfamide, epirubicin, and etoposide (IEV); under stable remission after HSCT. (ii) Refractory SLE-like condition treated with hydroxychloroquine, azathioprine, prednisone, high-dose steroids, methotrexate, mycophenolate mofetil, and anakinra. (iii) Start of rapamycin treatment since APDS2 diagnosis. MIP (Maximum Intensity Projection) Image of FDG PET/CT at staging (**a**), after transplantation (**b**,**c**) and after sirolimus treatment (**d**). Green arrows: hot bone marrow. Red arrows: hot lymph nodes. Yellow arrow: abnormal spleen metabolism. Values of the tracer uptake before and after treatment: BONE MARROW SUV max: 5.7 → 5 → 3.7 → below liver; SPLEEN SUV max: 4.3 → below liver → below liver → below liver; NODES SUV max: 6 → 4.7 → 8.4 → below liver. Abbreviations: SLE = systemic lupus erythematosus; auto-HSCT = autologous stem cell transplantation; APDS2 = activated phosphoinositide 3-kinase-δ syndrome 2.

An activating mutation of *PIK3R1* was found in a 30-year-old Romanian woman presenting to the Immunology Unit with a history of Hodgkin lymphoma, refractory systemic lupus erythematosus, autoimmune cytopenia, and diffuse lymphoproliferation [10]. As a child, she underwent adenotonsillectomy for hypertrophic tonsils. At 19 years of age, she was diagnosed with stage IIIA Hodgkin’s lymphoma, although atypical aspects emerged: pathohistological reexamination highlights the suspicion of a possible interfollicular HL-like picture in otherwise hyperplastic lymphadenopathy and FDG-PET/CT at baseline showing several hot supra and subdiaphragmatic lymph nodes with a very symmetrical distribution, located in the laterocervical region, axillary fossa, mediastinum at all levels and lomboaortic region. This pattern was associated with pathological inhomogeneous increased tracer uptake in axial and appendicular bone marrow (clearly higher than the normal liver uptake) and slight and diffuse tracer uptake in the spleen (Figure 1). She underwent several series of chemotherapy, although partial resolution of the lymphoproliferation was noticed. Symptomatic stable remission until the age of 30 was achieved following autologous stem cell transplant performed at 20 years of age. Nonetheless, FDG-PET/CT scans repeated after lymphoma treatment revealed an incomplete response, showing persistent diffuse increased and pathological FDG uptake in axial and appendicular bone marrow, although less intense. The lymph nodal uptake significantly improved, as progressive reduction in the number of hypermetabolic lymph nodes was noticed over time. However, refractory, enhanced tracer uptake was only found in the laterocervical region, again bilaterally and symmetrically. The spleen metabolism was normal, with a degree of uptake lower than the liver (Figure 1b,c). In addition, she developed clinical and laboratory manifestations that were interpreted as indicative of a systemic lupus erythematosus (SLE)-like condition: skin lesions, polyarthritis, polyserositis, recurrent episodes of fever, ANA test, ENA SS-A and anti-ds-DNA positivity, leukopenia, autoimmune hemolytic anemia, and immune-mediated thrombocytopenia. Disease manifestations were poorly responsive to numerous immunosuppressive therapeutic attempts throughout years of rheumatologic follow-up. At 29 years of age, the clinical picture was uncontrolled, showing typical features of macrophage activation syndrome-like systemic inflammation. Lung high-resolution computed tomography (HRCT) revealed diffuse altered ventilation, the presence of two solid nodules at the superior left lobe, mediastinal and right bronchial lymphadenopathies, and diffuse lymphoproliferation. FDG-PET/CT confirmed the persistence of refractory diffuse lymphoproliferation, with involvement of laterocervical, axillary, thoracic, gastric, iliac, and lower limb regions. Axillary lymph node biopsy and esophagogastroduodenoscopy were also performed, ruling out the diagnosis of lymphoma. Echocardiography documented left ventricular dilatation of unknown etiology, partly ascribed to chemotherapy. Since APDS2 diagnosis [9], immunoglobulin replacement therapy allowed a good control of infectious phenotype. Treatment with rapamycin was promptly started and the patient presented an impressive clinical improvement. FDG-PET/CT after one year of therapy with mTOR inhibitor confirmed complete normalization of the tracer distribution, with no signs of altered metabolism within lymph nodes and spleen. The axial bone marrow uptake reduced within normal limits, and appendicular bone marrow turned completely hypometabolic (Figure 1d) for the first time in 20 years of PET history.

## 3. Discussion

We report the case of a 30-year-old woman who received a late diagnosis of APDS2, showing some of the most common complications associated with this disease, including lymphoma, non-malignant lymphoproliferation, and immune dysregulation, before the start of the appropriate treatment [10]. Considering the growing knowledge of IEI, together with the advances in diagnostic and therapeutic fields, the lifespan of these patients is constantly increasing. A recent update remarked on the heterogeneity of APDS2 onset both in terms of age of presentation and of clinical manifestations. Although APDS2 frequently begins in childhood with increased infection susceptibility (especially to the herpes virus), growth retardation, tonsillar hypertrophy, autoimmunity, and other manifestations of immune-dysregulation, atypical or late-onset symptoms may occur. Refractory autoinflammatory or autoimmune manifestations, diffuse lymphoproliferation, gastrointestinal involvement, or malignancy could be found in adulthood. Interestingly, our case is one of the first reported adulthood forms of APDS2, with an age of onset of 19 years, and with apparent mild aspecific symptoms during childhood, except for tonsillar hypertrophy, different from the patients described so far [11]. It is worth underlining that pediatric medical history is often imprecise when collected in adulthood, especially if the patient comes from a foreign country, as in the case reported. Indeed, it is also possible that some pediatric clues are missed when the diagnosis is made in adults. Generally, before undergoing immunological evaluations, patients may be referred to many different specialists, often non-immunologists, for their complications or for therapy-resistant symptoms, with significant diagnostic delay and prognosis impairment. Therefore, given the morbidity and mortality associated with APDS2, it is important to expand awareness about IEI red flags, especially in non-immunological units and in adult clinics. A considerable gap exists between the broad scientific achievements and knowledge about IEI and the clinical practice. Importantly, adult medical services and non-immunological branches should carefully consider the hypothesis of a genetic disorder of the immune system underlying a complex immune-dysregulated phenotype (refractory or relapsing autoimmunity, infections, allergies, cancer susceptibility, autoinflammation, and lymphoproliferation), especially in young adults, referring the patient to a specialized center. 

Early diagnosis represents the key factor for patient survival and quality of life. Different targeted treatments have been developed and are available against potential IEI complications. Currently, APDS2 therapy is based on supportive treatment with antibiotic prophylaxis and immunoglobulin replacement against hypogammaglobulinemia, recurrent infections, and immune dysregulation. However, other specific treatments have been successfully tried to contrast disease evolution. Sirolimus, or rapamycin, known as a mammalian target of rapamycin (mTOR) inhibitor, acts by suppressing the hyperactivation of the PI3K/Akt/mTOR pathway and has been demonstrated to reduce lymphoproliferation in APDS2 patients, although non-lymphoproliferative manifestations were less responsive [4]. Hematopoietic stem cell transplantation (HSCT) represents the only curative treatment available up to now, as it reverses the disease phenotype [7]. Recent data showed a high incidence of graft failure, graft instability, and poor graft function both in APDS1 and APDS2 patients, worsened by the post-HSCT use of mTOR inhibitors. Therefore, strict evaluation of optimal timing, conditioning intensity, and patient status is essential, and more follow-up data are still needed to assess the length of phenotype reversal over time [8]. In any case, HSCT should indeed be considered a useful therapeutic option also in adulthood, for selected patients without relevant comorbidities and after appropriate multidisciplinary discussion. Specific targeted treatments for APDS1 and APDS2 are available. Selective PI3K δ inhibitors like leniolisib, nemiralisib, and seletalisib proved safe and effective against disease activity, although confirmatory studies and long-term evaluation are still needed [12]. The use of disease-attenuating agents like rapamycin or PI3K δ inhibitors may represent a beneficial bridge therapy to gain clinical stability and immunological control before HSCT. Recently, Rao et al. published the results of a phase 3, triple-blinded randomized trial (NCT02435173) conducted globally in patients over 12 years of age, showing good tolerance and efficacy, especially on lymphadenopathy and increase in circulating naïve B cells [13]. An open-label extension trial is underway to assess the long-term effects of leniolisib in this patient setting [14]. Moreover, the potential use of leniolisib in pediatric age is being investigated in two open-label, multicenter, single-arm studies conducted on children from 1 to 6 years and from 4 to 11 years of age with APDS. Given the broad spectrum of available therapeutic attempts for APDS, early recognition of this condition among IEI is crucial, as clinicians should aim to avoid organ disfunction and improve patient status before the onset of disease complications. 

Benign lymphoproliferation represents a hallmark of APDS2, involving most of the patients with a variety of clinical or subclinical manifestations. It can present either as chronic lymphadenopathy, hepatomegaly, splenomegaly, and mucosal infiltration, especially at ear-nose-throat and gastrointestinal levels. As reported in registries, histological specimens generally consist of atypical follicular hyperplasia, prominently composed of T-cells and small B-cell follicles, with ill-defined germinal centers and reduced or absent follicular mantle zones. T-cell subtypes commonly include programmed cell death protein 1, CD57, or both. In the intrafollicular area, numerous aggregates of large B cells can be observed [1,5]. Disseminated EBV and CMV-positive cells, or both, are frequently encountered, without aspects of infectious mononucleosis-like histology. Our patient experienced chronic lymphoproliferation, with hepatosplenomegaly and lymph node enlargement at laterocervical, axillary, mediastinal, gastric, iliac, and lower limb stations, not linked to chronic or uncontrolled EBV and CMV viremia. In addition, after the adequate treatment for lymphoma, she showed persistent diffuse FDG uptake at multiple lymphatic levels and bone marrow, atypical locations for HL, despite several cycles of chemotherapy. Lymph node biopsy ruled out a malignancy relapse. As a matter of fact, risk of neoplastic transformation is one of the major negative prognostic factors of this disease. APDS patients often have an history of multiple surgical interventions for recurrent lymphadenopathies. Currently, a standardized protocol to promptly evaluate and optimally manage non-malignant lymphoproliferation is lacking. The described case highlights the importance of carefully revised histopathological diagnoses of not-clear lymphoproliferative disorders performed in the past years to avoid misdiagnosis.

FDG PET/CT is a very sensitive, very safe (in terms of acute complications) and non-invasive imaging procedure commonly used in clinical practice to highlight the abnormal tracer uptake related to lymphomatous localizations. One advantage of this functional technique is that metabolic changes related to therapy efficacy occur much earlier than morphological changes, so that the normal diagnostic flow chart includes FDG PET/CT not only to stage the disease but also to assess therapy response at the beginning and at its completion, and to demonstrate an early relapse during follow-up. Furthermore, FDG PET/CT is used to evaluate bone marrow lymphomatous involvement. In an IEI setting, this imaging method has been studied for the monitoring of lymphadenopathies in Autoimmune Lymphoproliferative Syndrome (ALPS). Carrasquillo et al. found a visual and quantitative overlap in FDG uptake between ALPS patients with non-malignant and malignant lymphoproliferation, but they affirmed that FDG-PET/CT can play a role in guiding the choice of biopsy site due to its high sensitivity [15]. In another patient with ALPS, FDG-PET/CT was used to monitor response to treatment with rituximab by evaluating metabolic activity changes of involved lymph nodes [16]. Based on these well-known assumptions, this technique may also be interesting for studies of other hypermetabolic diseases of the immunological system, such as APDS2. Of course, the increased tracer uptake is somehow an aspecific sign, but once the diagnosis is carried out and a particular pattern is identified, like in our case, the exceptional sensitivity of this technique could ideally be exploited to confirm the therapeutic efficacy. In this patient, initial inadequate response to lymphoma treatment, due to the coexistence of diffuse lymphoproliferation, led to the need of numerous imaging studies and vain treatment attempts before the diagnosis of IEI. One drawback of FDG-PET/CT is certainly related to the use of radioactivity. Being a pediatric disease, the radiation exposure represents an issue, especially in younger patients. Overall, with a state-of-the-art tomograph, the level of absorbed radiation is within an “intermediate exposure” (higher than conventional X-rays but lower than a total body diagnostic CT), which implies an optimized and planned indication to the scan in any case. However, large new field-of-view scanners, which are already on the market, will allow a further dramatic reduction in tracer-injected activity to obtain a diagnostic scan, leading this procedure towards a much wider employment in the pediatric imaging of the near future.

## 4. Conclusions

Awareness on IEI in clinical settings is of the utmost importance, as early diagnosis and rapid access to treatment before the onset of comorbidities are the key factors to improve patient prognosis. One of the major challenges is the lack of specific guidelines for the diagnosis of non-malignant lymphoproliferation. Our case suggests that FDG-PET/CT may represent a useful and non-invasive tool in differentiating malignant proliferation from immune dysregulation phenotypes. Firstly, the presence of atypical patterns and unusual metabolic uptake at FDG-PET/CT should be interpreted as a red flag for the need of an early immunological evaluation, especially in patients with other features of immune dysregulation. In addition, FDG-PET/CT shows promising results for the assessment of the response to therapy, thanks to the more rapid metabolic modifications compared to morphological changes, and its use should indeed be considered during the follow-up of these patients. Although more extended studies are needed to validate the efficacy and safety of this method and its application with the new available therapies, our case confirms the potential role of FDG-PET/CT in APDS2, as a non-invasive technique for the management of the wide spectrum of lymphoproliferation associated with IEI.

## Data Availability

No new data were created or analyzed in this study. Data sharing is not applicable to this article.

## References

[1] Elkaim E., Neven B., Bruneau J., Mitsui-Sekinaka K., Stanislas A., Heurtier L., Lucas C.L., Matthews H., Deau M.C., Sharapova S. (2016). Kracker, Clinical and immunologic phenotype associated with activated phosphoinositide 3-kinase δ syndrome 2: A cohort study. J. Allergy Clin. Immunol..

[2] Al-Shami A., Naccache P.H. (1999). Granulocyte-Macrophage Colony-stimulating Factor-activated Signaling Pathways in Human Neutrophils: Involvement of Jak2 in the stimulation of phosphatidylinositol 3-KINASE. J. Biol. Chem..

[3] Kämpe M., Lampinen M., Stolt I., Janson C., Stålenheim G., Carlson M. (2012). PI3-Kinase Regulates Eosinophil and Neutrophil Degranulation in Patients with Allergic Rhinitis and Allergic Asthma Irrespective of Allergen Challenge Model. Inflammation.

[4] Maccari M.E., Abolhassani H., Aghamohammadi A., Aiuti A., Aleinikova O., Bangs C., Baris S., Barzaghi F., Baxendale H., Buckland M. (2018). Disease evolution and response to rapamycin in activated phosphoinositide 3-kinase δ syndrome: The European society for immunodeficiencies-activated phosphoinositide 3-kinase δ syndrome registry. Front. Immunol..

[5] Coulter T.I., Chandra A., Bacon C.M., Babar J., Curtis J., Screaton N., Goodlad J.R., Farmer G., Steele C.L., Leahy T.R. (2017). Cant, Clinical spectrum and features of activated phosphoinositide 3-kinase δ syndrome: A large patient cohort study. J. Allergy Clin. Immunol..

[6] Thouenon R., Moreno-Corona N., Poggi L., Durandy A., Kracker S. (2021). Activated PI3Kinase Delta Syndrome—A Multifaceted Disease. Front. Pediatr..

[7] Notarangelo L.D. (2019). Hematopoietic stem cell transplantation for activated phosphoinositide 3-kinase δ syndrome: Who, when, and how?. J. Allergy Clin. Immunol..

[8] Dimitrova D., Nademi Z., Maccari M.E., Ehl S., Uzel G., Tomoda T., Okano T., Imai K., Carpenter B., Ip W. (2022). International retrospective study of allogeneic hematopoietic cell transplantation for activated PI3K-delta syndrome. J. Allergy Clin. Immunol..

[9] Barrington S., Barrington S.F., Trotman J. (2021). The role of PET in the first-line treatment of the most common subtypes of non-Hodgkin lymphoma. Lancet Haematol..

[10] Conti F., Catelli A., Cifaldi C., Leonardi L., Mulè R., Fusconi M., Stefoni V., Chiriaco M., Rivalta B., Di Cesare S. (2021). Case Report: Hodgkin Lymphoma and Refractory Systemic Lupus Erythematosus Unveil Activated Phosphoinositide 3-Kinase-δ Syndrome 2 in an Adult Patient. Front. Pediatr..

[11] Staels F., Collignon T., Betrains A., Gerbaux M., Willemsen M., Humblet-Baron S., Liston A., Vanderschueren S., Schrijvers R. (2021). Monogenic Adult-Onset Inborn Errors of Immunity. Front. Immunol..

[12] Berglund L.J. (2023). Modulating the PI3K Signalling Pathway in Activated PI3K Delta Syndrome: A Clinical Perspective. J. Clin. Immunol..

[13] Rao V.K., Webster S., Annašediv A.A., Annašedivá A., Plebani A., Schuetz C., Shcherbina A., Conlon N., Coulter T., Dalm V.A. (2023). A randomized, placebo-controlled phase 3 trial of the PI3Kδ inhibitor leniolisib for activated PI3Kδ syndrome. Blood.

[14] Newman H., Teachey D.T. (2023). PI3king apart a rare disease with targeted therapy. Blood.

[15] Carrasquillo J.A., Chen C.C., Price S., Whatley M., Avila N.A., Pittaluga S., Jaffe E.S., Rao V.K. (2019). ^18^F-FDG PET Imaging Features of Patients with Autoimmune Lymphoproliferative Syndrome. Clin. Nucl. Med..

[16] Cistaro A., Pazè F., Durando S., Cogoni M., Faletti R., Vesco S., Vallero S., Quartuccio N., Treglia G., Ramenghi U. (2014). Autoimmune lymphoproliferative syndrome and non-Hodgkin lymphoma: What ^18^F-fluorodeoxyglucose positron emission tomography/computed tomography can do in the management of these patients? Suggestions from a case report. Rev. Esp. Med. Nucl. Imagen Mol. (Engl. Ed.).

